# Ambient and Wearable Sensor Technologies for Energy Expenditure Quantification of Ageing Adults

**DOI:** 10.3390/s22134893

**Published:** 2022-06-29

**Authors:** Alessandro Leone, Gabriele Rescio, Giovanni Diraco, Andrea Manni, Pietro Siciliano, Andrea Caroppo

**Affiliations:** National Research Council of Italy, Institute for Microelectronics and Microsystems, 73100 Lecce, Italy; gabriele.rescio@cnr.it (G.R.); giovanni.diraco@cnr.it (G.D.); andrea.manni@le.imm.cnr.it (A.M.); pietro.siciliano@le.imm.cnr.it (P.S.); andrea.caroppo@cnr.it (A.C.)

**Keywords:** ageing adults, energy expenditure, posture classification, ambient sensor, wearable sensor, AAL

## Abstract

COVID-19 has affected daily life in unprecedented ways, with dramatic changes in mental health, sleep time and level of physical activity. These changes have been especially relevant in the elderly population, with important health-related consequences. In this work, two different sensor technologies were used to quantify the energy expenditure of ageing adults. To this end, a technological platform based on Raspberry Pi 4, as an elaboration unit, was designed and implemented. It integrates an ambient sensor node, a wearable sensor node and a coordinator node that uses the information provided by the two sensor technologies in a combined manner. Ambient and wearable sensors are used for the real-time recognition of four human postures (standing, sitting, bending and lying down), walking activity and for energy expenditure quantification. An important first aim of this work was to realize a platform with a high level of user acceptability. In fact, through the use of two unobtrusive sensors and a low-cost processing unit, the solution is easily accessible and usable in the domestic environment; moreover, it is versatile since it can be used by end-users who accept being monitored by a specific sensor. Another added value of the platform is the ability to abstract from sensing technologies, as the use of human posture and walking activity for energy expenditure quantification enables the integration of a wide set of devices, provided that they can reproduce the same set of features. The obtained results showed the ability of the proposed platform to automatically quantify energy expenditure, both with each sensing technology and with the combined version. Specifically, for posture and walking activity classification, an average accuracy of 93.8% and 93.3% was obtained, respectively, with the wearable and ambient sensor, whereas an improvement of approximately 4% was reached using data fusion. Consequently, the estimated energy expenditure quantification always had a relative error of less than 3.2% for each end-user involved in the experimentation stage, classifying the high level information (postures and walking activities) with the combined version of the platform, justifying the proposed overall architecture from a hardware and software point of view.

## 1. Introduction

The world population continues to grow older rapidly, as fertility rates have fallen to very low levels in most world regions and people tend to live longer. From 2025 to 2050, the older population is projected to almost double to 1.6 billion globally, whereas the total population will grow by just 34% over the same period [1]. An increased life expectancy is certainly an opportunity, but it also has negative health aspects, such as physical, mental and cognitive deterioration [2,3]. In the last two years, the new coronavirus (SARS-CoV-2) has significantly changed the lifestyle of the world’s population, not only in terms of lives lost but especially from an economic and social point of view. COVID-19 has had a very strong impact, especially on daily routines, due to restrictions put in place by various governments that forced people to stay at home in isolation for very long periods [4,5,6]. In this context, scientists have investigated in the direction of designing and implementing easy-to-use technological platforms/solutions to monitor specific behaviors directly at the home of the observed subject. It is obvious that such monitoring is more required for fragile subjects and ageing adults. Consequently, there was an effort by the research communities to increase the availability of different services and devices to the elderly through dedicated technologies installed in the so-called “intelligent” home. This vision relies on the potentials of pervasive Information and Communication Technology (ICT) to make the house environment adaptable to the users’ needs in order to transform it into an intelligent environment. For example, ambient and wearable sensors combined with automatic behavioral analysis solutions create a living environment adaptable to the characteristics of end-users [7]. In addition, it is worth highlighting that, in the Internet Of Things (IOT) sector, research activities with the aim of providing automated tools for the development of healthcare systems based either exclusively on Body Sensor Networks (BSNs) [8] or on heterogeneous sensor systems [9] are increasingly common. One of the most significant applications of this paradigm is Ambient Assisted Living (AAL), allowing the elderly to live independently in their houses for as long as possible, delaying hospitalization in the last part of their life and generally improving their quality of life through personalized healthcare [10].

In this application field, it is very important to monitor the temporal trend of Physical Activity (PA) and to quantify its level in an objective way in order to have an indicator for the possible onset of pathologies, as it has been demonstrated that the lack of motor activity can lead to chronic health disorders [11]. A PA evaluation, including the type, intensity and duration of activities, is very important to prevent and/or correct bad habits. In addition, the time spent in sedentary behavior grew significantly during the pandemic period. It is widely known that a sedentary life-style is a risk factor for metabolic syndrome or obesity [12,13], and independently of PA levels [14]. All this leads to the conclusion that an objectively monitoring of PA and the associated estimates of Energy Expenditure (EE) can provide important feedback, allowing for a person to regulate and modify the level of PA and avoid having a sedentary lifestyle in order to maintain their physical well-being.

EE refers to the amount of energy an individual uses to maintain essential body functions (respiration, circulation, digestion) and is a result of physical activity. The total daily energy expenditure is determined by the resting or Basal Metabolic Rate (BMR), food-induced thermogenesis, and energy expended as a result of physical activity.

EE can be measured in different ways. The gold standard is direct calorimetry, which measures the actual heat emitted by the human body during activity or rest, in a special room (room calorimeter) [15]. Another methodology for EE quantification is indirect calorimetry [16], which measures the concentration of inhaled and exhaled gases. The consumption of oxygen (O_2_) and the production of carbon dioxide (CO_2_) can be converted into EE Weir’s equation [17]. Indirect calorimetry is one of the most used techniques due to the existence of portable indirect calorimeters. Given the practical limitations of room calorimeters and the cost of portable indirect calorimeters, several solutions have been developed to estimate the EE through the PA analysis and using commercial sensors.

This work describes the design and implementation of a platform capable of automatically quantifying the EE of a subject. This objective measurement is implemented using commercial and low-cost ambient and wearable sensors and by an inexpensive processing unit. The idea of using heterogeneous sensors is motivated by the fact that, in this way, we expand the number of end-users, as they may accept only a certain type of sensor technology.

The main contributions of the proposed work are listed below:

- An algorithmic framework for the classification of postures and walking activity using commercial ambient and wearable sensors is designed and implemented;
- A data fusion algorithmic block is implemented on a low-cost processing unit;
- Three different machine learning classification algorithms are compared to distinguish between posture and walking activitiy after data fusion;
- EE was objectively quantified for each end user through the time trend of the postures and the walking activity length via the lookup table of the corresponding MET values.

The remainder of this paper is structured as follows. Section 2 reviews the related work. Section 3 reports an overview of the overall architecture of the proposed platform and a detailed description of computational framework for human posture and walking activity recognition in correspondence with each sensor technology. The same section provides some information about the methodology designed and implemented for EE quantification. Participant characteristics, data collection, experimental setup and results are presented in Section 4. Finally, Section 5 shows both our conclusions and discussions on some ideas for future work.

## 2. Related Work

Sensor technologies can be divided into two types: ambient and wearable. In a contact-based sensor system (wearable), users must wear devices on body segments to measure EE. The main advantage of contact-based systems is their suitability for outdoor activities. However, wearing many sensors when performing PA is impractical and, moreover, the scientific literature has demonstrated in many works that the position of a wearable sensor greatly affects the accuracy of EE estimation. Moreover, the battery life of wearable devices is a critical challenge.

Instead, non-contact approaches (based on an ambient sensor) have been proposed to solve the problems of using wearable devices to estimate EE. This last category of sensors, however, is subject to a lower acceptability, especially by the elderly, who see their privacy violated. Moreover, EE quantification can be distorted by an incorrect recognition of human postures and/or activities due to possible occlusions between the vision system and the user.

The following is a brief state of the art pertaining to scientific works in which the two sensory technologies have been used for EE quantification.

For example, a commercial device widely diffused and capable of measuring different aspects of human behavior is the activPAL^TM^ (ACT) (PAL Technologies Ltd., Glasgow, UK). ACT is a small lightweight electronic device worn under clothing, attached directly to the skin on the midline of the anterior area of the thigh. ACT is qualified to detect posture based on thigh acceleration, including the gravitational factor. Proprietary algorithms (intelligent activity classification) are used to classify time as standing, sitting, lying down and stepping. Moreover, ACT estimates EE and provides information on the number of steps taken, rhythm and sit-to-stand and stand-to-sit transitions. ACT has been shown to be a solid and valid analyzer of step counts in the elderly [18,19].

In [20], the authors compared differences in EE across three different postures: sitting, standing and lying down. Moreover, they determined the associations between the change in EE across the previous postures with anthropometric and body composition parameters in young healthy adults. The EE measurements were performed by indirect calorimetry following the recommendations reported in [18]. They demonstrated that standing increases EE above sitting and lying values (~10%), whereas sitting and lying paradoxically seem to represent similar EE. Taken together, these findings suggested that decreasing lying and sitting times could be a simple strategy to slightly increase EE.

Another interesting developed solution for the approximation of PA and subsequent EE quantification is reported in [21], where the authors used the SenseWear Armband (SWA) that permits collecting a diversity of physiological signs and integrates a bi-axial accelerometer, a galvanic skin resistance sensor and a body temperature sensor. Generally, the SWA is used for an accurate EE estimation and step count during treadmill effort, providing a reasonably accurate measure of step count. However, the results reported in the work have also demonstrated that the SWA permits quantifying, with a sufficient accuracy level, the amount of PA, providing a methodology for amn automatic decision-making system for the increasing of activity in aged people. A limitation of the work is that, while armbands technologies have proven to be fine devices for tasks of daily life (or low to moderate activity), they have not been appropriate for higher intensity exercise, so the usage of the commercial device for the evaluation of physical and sedentary levels is not ideal.

On the other hand, the work proposed in [22] described a wearable system consisting of a shoe equipped with a smart sensor and a mobile phone for signal processing, pattern recognition and real-time user feedback of expended calories and other PA information. The authors highlighted that the use of logistic discrimination or a multilevel perceptron instead of a supervised classifier such as a Support Vector Machine (SVM) reduced the execution time and memory requirements by a factor of >10${}^{3}$, maintaining a comparable accuracy of classification and EE estimation. Moreover, the overall high accuracy of EE estimation in four different tested EE models has also pointed out the benefits of the proposed wearable system relying on in-shoe sensors. A limitation of this study is that the EE of the subjects was measured under a specific (although randomized) activity protocol, but, as affirmed by the authors, the number of activities in free living is much greater and not so clear cut. Another important limitation is that the study was conducted on a population mostly consisting of young adults, not involving elderly people in the experimentation, for whom, there is more need to monitor PA and EE.

In a very recent work [23], a comparison of the Absolute Error Rate (AER) of EE measured by the wrist-worn and the hip-worn ActiGraph GT3X over a 24 h period in free-living conditions in young and older adults was reported. Obtained results demonstrated that EE was overestimated when measured by the wrist-worn activity trackers. Furthermore, they found a significant negative correlation between the AER and EE provided by the hip-worn activity tracker in the overall population. Finally, they also reported an effect of age on the AER, with a lower AER for young participants.

As for ambient sensors only, there are few scientific papers in the literature in which this type of device is used to quantify EE. Nathan et al. [24] estimated the mechanical work performed by the human body and estimated subsequent metabolic energy using predictive algorithmic models and a Kinect sensor. They achieved the objective of EE quantification through the following steps: (1) capturing the mechanical work and metabolic cost for a range of exercises of varying intensity and movement type; (2) deriving biomechanically appropriate features from mechanical work; (3) building a predictive multivariate model using nonparametric regression based on the derived features. The results reported in the work demonstrated that, for high-energy activities, such as standing or jumps, EE estimation can be made accurately, but that, for low-energy activities, the posture of static poses should be considered as a contributing factor. In [25], the authors developed a noncontact method for EE estimation by using a camera and classical image processing approaches. However, the EE estimation error reported in the study was high.

The work of Yang et al. [26] is focused instead on the use of a smartphone camera and on the development of algorithms for the analysis of body movement and the body’s effort. The realized system is able to objectively assess the intensity and EE of popular indoor workouts, including sit ups, push ups, jumping jacks and squats. In this work, the algorithm is based on a hierarchical kinematic approach that analyzes the body movement (and subsequent EE) in terms of different layers, each with an increasing level of details. The authors compared the results obtained with the EE values assessed with the gold standard indirect calorimetric method.

A very interesting work is described in [27], where the authors introduced a framework for EE estimation from RGB-D data in a living room environment. They implemented a cascaded and recurrent approach that explicitly detects activities as an intermediate to select type-specific mapping functions for a final calorific estimation. A very important contribution of this research activity is the introduction of a dataset (called SPHERE calorie) linking more than 10 h of RGB-D video data to ground truth calorie readings from indirect calorimetry based on gas exchange.

The simultaneous use of ambient and wearable sensors is receiving a great deal of attention in specific areas of research, such as robotics [28], action or gesture recognition [29,30] and AAL applications such as, for example, human behavior understanding, fall detection and remote health monitoring [31,32]. Although the involvement in a platform of heterogeneous sensors has the advantage of complementing shortcomings of individual modalities, wearing a multitude of sensors or being monitored 24 h a day through a vision sensor can cause user acceptance issues.

## 3. Materials and Methods

**Figure 1:**
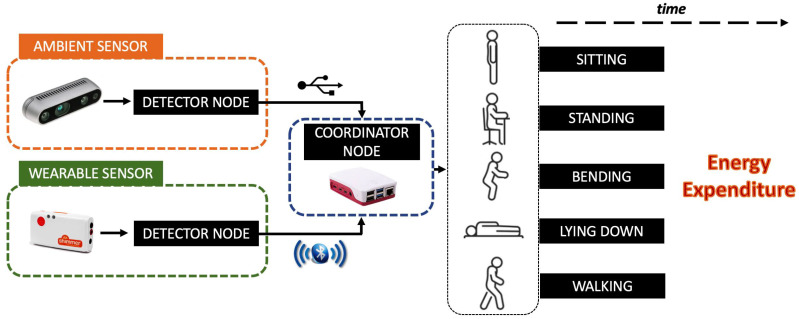
Schematic representation of the proposed platform for EE quantification. Two different sensor technologies (ambient and wearable) transmit high-level information (e.g., posture label with timestamp) to a coordinator node.

The overall architecture of the proposed platform is depicted in Figure 1. It has a hierarchical network topology, compounded by two detector nodes that manage, respectively, an ambient sensor node and a wearable sensor node. These nodes provide high-level information to a coordinator node. The use of the camera makes it possible to cover any detection deficiencies exhibited by wearable sensors. Conversely, wearable sensors make it possible to compensate for camera detection shortcomings, i.e., in the presence of occluding objects (e.g., table, bed, etc.). In addition, using a 3D camera (stereoscopic in the specific case) allows for resolving situations of perspective ambiguity. From the hardware point of view, all of the components involved in the actual version of the platform were selected to meet typical requirements of AAL applications. The computational framework comes with features that allow for an easy integration into larger AAL systems. In fact, it was conceived as a distributed, modular and open architecture implemented by coordinator and detector nodes.

### 3.1. Computational Frameworks for Human Posture and Walking Activity Recognition

#### 3.1.1. Ambient Sensor

**Figure 2:**
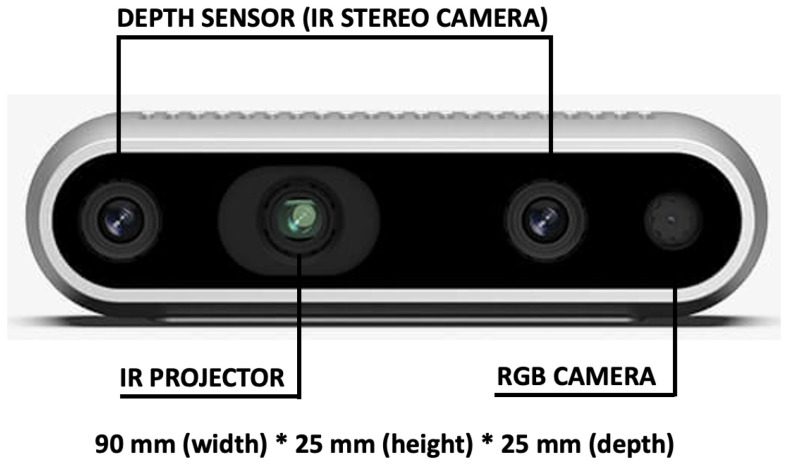
Intel^®^ RealSense^TM^ model D435i. It integrates a RGB sensor, a stereo image sensor and an infrared projector for the depth data collection.

The ambient sensor used in the actual version of the platform was the commercial and low-cost RealSense^TM^ D435i camera [33] produced by Intel^®^ (Figure 2). It integrates: the latest Intel^®^ RealSense^TM^ Vision Processor D4 to handle the complex depth algorithm, an RGB sensor to collector color data, a stereo image sensor to capture and calculate disparity between images and, finally, an infrared projector to illuminate objects and collect depth data.

The powerful vision processor uses 28 nanometer (nm) process technology and supports up to 5 MIPI Camera Serial Interface and 2 channels to compute real-time depth images and accelerate output, generating up to 90 frames per second (fps) in a depth video stream. In addition, it integrates an advanced stereo depth algorithm and a new design for more accurate depth perception and longer range. With the optimal calibration, the stereo depth perception has an error rate as low as 1%. In the optimal environment, this camera can capture data from a distance as far as 10 m in both indoor and outdoor environments. In addition, with the global image shutter and wide field of view (69.4 × 42.5 × 77${}^{\circ }$), the Intel^®^ RealSense^TM^ Depth Camera D435i offers the capability to capture and stream the depth data of moving objects effectively, providing high depth perception accuracy. Postures and walking activity were estimated positioning the camera on a tripod at 143 cm from the floor.

**Figure 3:**
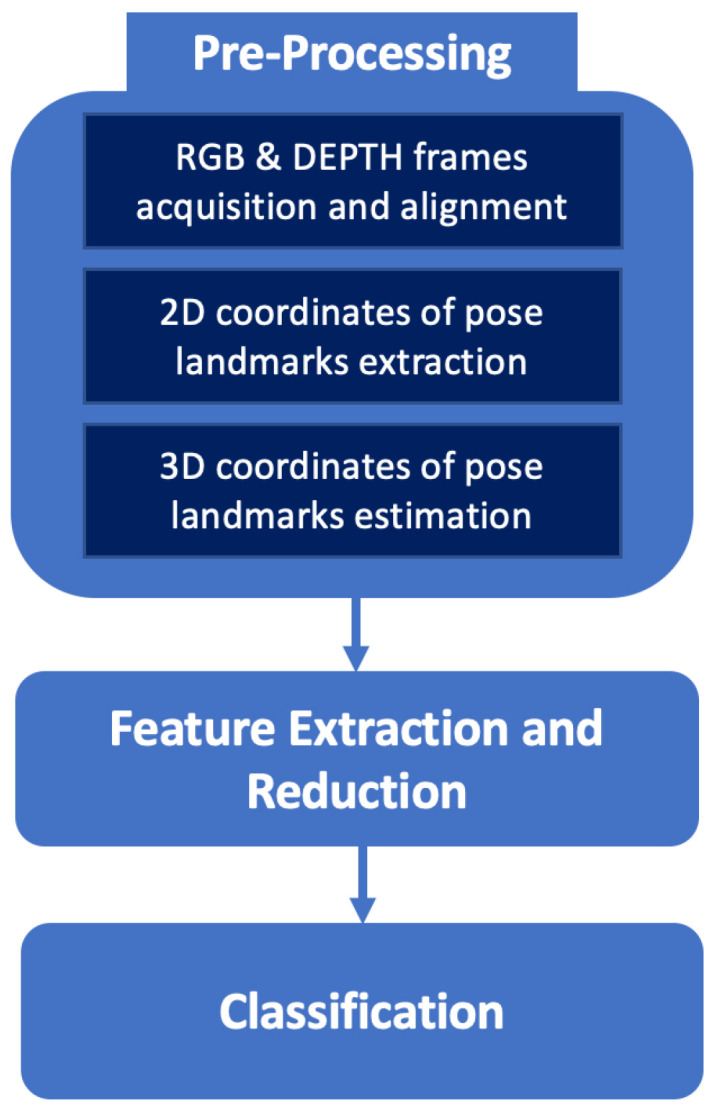
Proposed pipeline for posture and walking activity classification using an ambient sensor. It consists of a pre-processing of the acquired images followed by a feature extraction and reduction step and, at last, a classification block returning four different postures and walking activity at different speeds.

The computational framework for posture estimation using the 3D camera consists of the following functional blocks, as shown in Figure 3:

- Acquisition of RGB and depth frame from the 3D camera and their alignment;
- Extraction of the pose landmarks from the RGB frame;
- Estimation of the 3D coordinates of the pose landmarks from the depth frame;
- Definition, extraction and reduction in postural features;
- Posture and walking activity classification.

**Figure 4:**
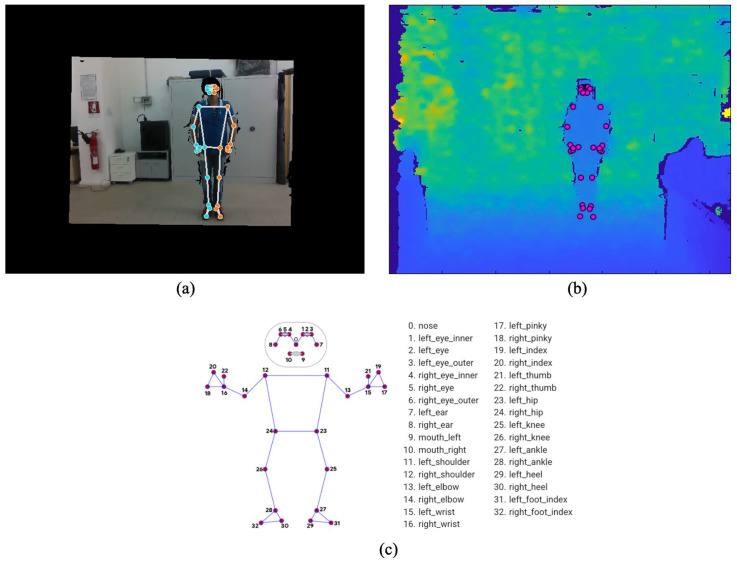
The RGB (**a**) and depth (**b**) frames and, then, the 33 landmark BlazePose model (**c**) used to define postural features.

The RGB and depth frames were acquired using the library supplied with the RealSense^TM^ D435i camera in Python language. After the acquisition, the frames were aligned using the same library to match the coordinates of corresponding points between the RGB and depth image planes, as shown in Figure 4a,b. The library also provides the function for calculating the 3D coordinates starting from the 2D coordinates in the image plane of the depth frame.

Regarding the postural features, a model-based approach was used to adapt a skeleton composed of various pose landmarks of the body of the monitored subject. In order to estimate the pose model, the open-source framework MediaPipe [34] was used in the form of a Python library. MediaPipe provides a pipeline based on Machine Learning (ML) and Deep Learning (DL) consisting of three independent models for estimating the monitored subject’s pose, face and hands. Each model uses its input frame for real-time capture of the video stream.

The MediaPipe pose detector, called BlazePose, was used to define the postural features in this study. Using this pose model, it is possible to identify 33 pose landmarks, as shown in Figure 4c, from each RGB frame (aligned with the corresponding depth frame). It is important to note that this model was optimized to achieve real-time performance on mobile devices in Python. In particular, the model uses a two-step pipeline, which detects the region of interest of the person in the RGB frame and re-crops the frame to predict the pose landmarks. Then, the 33 pose landmarks estimated by BlazePose on the RGB frame were transformed into 3D coordinates using the specific function of the RealSense^TM^ library, providing, as input, the corresponding aligned depth frame and the intrinsic parameters of the RealSense^TM^ D435i camera.

Given the 3D coordinates of the 33 pose landmarks and the total height of the subject, a 100-dimensional feature space was obtained. A specific study was conducted in order to reduce the dimensionality of the feature space, optimizing, in this way, the processing performance on devices of limited capacity. Observing that the pose landmarks relative to the body are positioned at the joint of the limbs and near the extremities of the torso, the first reduction in dimensionality was obtained by assuming the distance between the pose landmarks (i.e., junction nodes and extremities of the torso) as constant and considering only the angles subtended by consecutive segments.

Subsequently, the classification performances (in terms of accuracy) and the computational load (in terms of execution time) were evaluated in correspondence with different combinations of segments and angles. The feature analysis indicated that the best tradeoff was to consider segments (more precisely, their angles) that join the head, torso and legs, with the addition of the overall height of the monitored subject. A further reduction in the features was obtained by combining the following pairs of pose landmarks A = (9, 10), B = (11, 12), C = (23, 24), D = (25, 26), E = (27, 28) (see Figure 4c), substituted by the midpoint of their 3D coordinates. The further analysis step made it possible to reduce the junction nodes, ultimately maintaining only the midpoints A, C and D.

Posture and walking activity classification represents the last block of the framework, implemented using a multi-class classifier of the SVM type [35]. SVM can be considered a technique that uses a linearly separated hypothesis space in a multi-dimensional feature space, trained using a learning algorithm based on optimization theory and derived from statistical learning theory. SVM was initially developed to model separation hyperplanes for classification problems. Subsequently, SVM was generalized to construct nonlinear separation functions for real-valued approximation functionals. To make the framework computationally light, a polynomial-type SVM classifier of degree equal to three and kappa parameter equal to 0.61 was adopted in this study, determined by exhaustive research.

#### 3.1.2. Wearable Sensor

The wearable system consists of an elastic band integrating the Shimmer3 IMU inertial device [36], which is equipped with the following sensors:

- Tri-axial accelerometer;
- Magnetometer;
- Pressure and temperature sensor;
- Tri-axial gyroscope.

**Figure 5:**
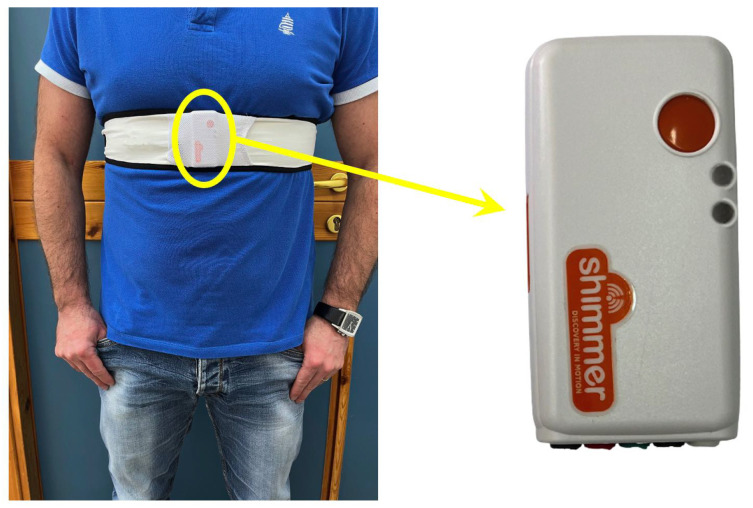
Wearable system integrating an elastic band (**left**) and Shimmer3 IMU inertial device (**right**) equipped with the following sensors: tri-axial accelerometer, magnetometer, pressure and temperature sensor and tri-axial gyroscope.

In order to recognize postures and walking activity over time, attention was focused only on the analysis of signals from the triaxial accelerometer, as they allow for good performance for motion analysis with low computational cost. The Shimmer3 accelerometer is DC coupled, so it is possible to evaluate both accelerations in static and dynamic conditions along the three axes. The device features a low-power wireless Bluetooth connection for non-invasive data transmission. The life duration of its battery is approximately 8 h in streaming mode. The data were acquired with a sampling frequency of 50 Hz, which is enough to evaluate human postures. The data are in the decimal format and represent the acceleration values with full scale in the range of ±2 g. The utilized wearable system is shown in Figure 5: the band allows for an optimal adherence and stability of the device to the chest, reducing noise on the signal due to improper movements of the device. It was decided for the sensor to be placed on the chest because accelerometers in that position have been proven to be better for posture recognition according to [37].

**Figure 6:**
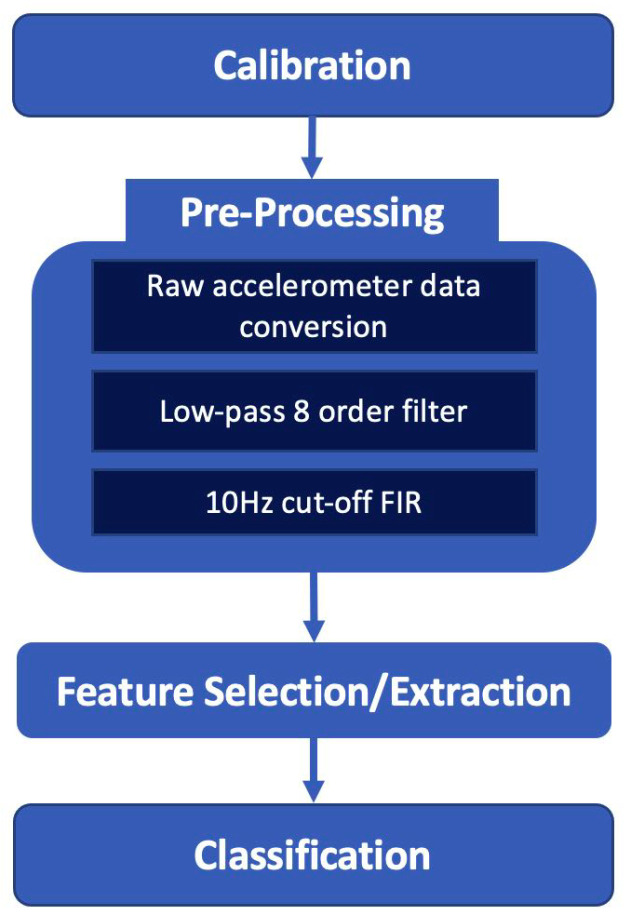
Proposed pipeline for posture and walking activity classification using a wearable sensor. It consists of a calibration stage to verify that the device was worn correctly, pre-processing of the acquired accelerometer signals followed by a feature selection/extraction step and, at last, a classification block returning four different postures and walking activity at different speeds.

The acceleration data on three axes were sent to a Raspberry Pi 4 and processed with a software developed in Python programming language. The most relevant phases of the software framework are summarized in Figure 6.

In the pre-processing step, the data were converted into gravitational units to represent acceleration data in the range of ±2 g; in this way, it is possible to extract the angle of the user posture with respect to the vertical direction, as described in [38]. Then, the samples were filtered out by a low pass 8-order, 10 Hz cut-off Finite Impulse Response (FIR) filter to reduce the noise due to environment and human tremor.

Regarding the calibration phase, it was introduced to verify that the device was worn correctly and to calculate the initial conditions necessary for data processing. Calibration is performed whenever the user wears the device. The calibration process has a duration of 30 s and memorizes the user’s static acceleration values on the three axes while the user is in a motionless and standing position. If the acquired acceleration values are within a predefined tolerance range, the calibration is successful and the next step of feature extraction can be performed.

The data thus processed were used for the feature extraction phase. The purpose of this phase is to obtain relevant information from the accelerometric signals useful for posture assessment. Several time domain and time–frequency domain features utilized in medical and technical applications for monitoring the human posture were investigated for this study [39]. Through the Lasso feature selection method [40], the following features were chosen: average, energy, dynamic and static acceleration variation, kurtosys and skewness for each axes. The size of the sliding window was set to 300 ms, with an incremental window of 50 ms.

Finally, for the classification, a supervised approach was adopted. In particular, SVM, K-Nearest Neighbors (KNN) and Random Forest (RF) were tested and the best performing were obtained by using the RF classifier. The RF algorithm [41] creates a collection of predictors from a set of decision trees that are produced at random in datasets. It represents a decision tree in terms of hyper-parameters. To classify the input vector, each classifier is constructed using a vector that is independent of the input vector, and each tree votes for the largest number of classes. RF adds more randomness to the model while increasing the trees. It detects the best feature in a random subset of features. In our approach, the number of estimators in the forest was fixed to 29, whereas the maximum tree depth was set to 26.

#### 3.1.3. Elaboration Unit

**Figure 7:**
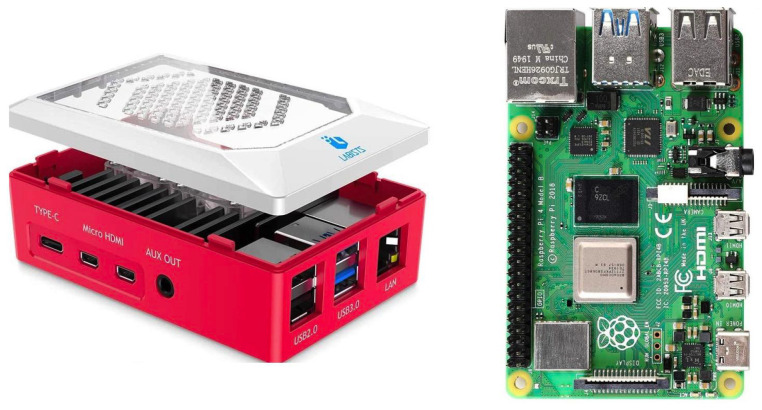
Elaboration unit (Raspberry Pi 4 Model B) for the acquisition and processing of sensory data and the fusion of high-level information classified by sensor nodes. Used case on the left and electronic board on the right.

To favor a wide diffusion of the proposed solution, the system presented in this work foresees the involvement of a low-cost processing unit, easily available on the market and generally used for the development of open platforms. Raspberry Pi 4 Model B (Figure 7) is the latest product in the popular Raspberry Pi computer versions. Processor speed, memory, connectivity and multimedia performance are better than previously released Raspberry Pi versions. The Raspberry Pi Foundation provides Raspbian, a Debian-based Linux distribution for download. It has Broadcom BCM2711, quad-core Cortex-A72 (ARM v8), 64-bit 1.5 GHz processor; 1 GB, 2 GB or 4 GB LPDDR4 (depending on model) memory; LAN, Bluetooth 5.0, Gigabit Ethernet, 2 USB 3.0 and 2 USB 2.0; 40 general-purpose input/output (GPIO) pins and a micro SD card slot for loading operating system and data storage [42].

As for interfacing with the sensors, the wearable sensor was connected to Raspberry via Bluetooth protocol, whereas the ambient sensor required a wired USB connection.

Algorithms for the acquisition and processing of sensory data were implemented on the elaboration unit, as well as logics for the management of the fusion of high-level information classified by the sensory nodes.

#### 3.1.4. Data Fusion

As illustrated in the previous sub-sections, both the ambient sensor and the wearable sensor were used for automatic recognition of four different human postures and walking activities at different speeds. It is well known that there are recognition rate limitations when using a single modality sensor, as no single mode can address all of the issues that occur in the real-world setting. In our case, for example, the difficulty in classifying standing and sitting postures with the wearable sensor was managed using the ambient sensor, which, however, presents difficulties in classifying postures in the presence of occlusions, but this last problem was not present in the wearable sensor. Therefore, the simultaneous involvement of the two technologies may allow them to compensate for their shortcomings and improve total recognition performance. Although each sensor technology can operate independently, a data fusion scheme is required to merge the information coming from each subsystem, thus improving the reliability of the overall platform. For this reason, the proposed platform for EE quantification was accompanied by a coordinator node (see Figure 1), which has the task of integrating algorithmic logics for the fusion of high-level information received from the ambient and wearable detector node. Several techniques have been developed over the years to fuse different data modalities for posture and activity recognition. While data fusion is a very broad topic, in the present work, two very specific techniques were considered: (a) decision-level fusion, and (b) feature-level fusion. Decision-level fusion, or fusion of classifiers, consists of processing the classification results of prior classification stages. The main goal of this procedure is to take advantage of the redundancy of a set of independent classifiers to achieve higher robustness by combining their results [43]. On the other hand, a feature-level fusion scheme integrates unimodal features before learning concepts [44]. The two main advantages of this scheme are the use of only one learning stage and taking advantage of mutual information from data.

From the analysis of the application context described in this paper and considering the type of high-level features used for EE quantification obtained from each sensing technology, it is beyond doubt that decision-level fusion technique has a main point of weakness, which is that, if the data of one sensor are missing, then its full capabilities cannot be exploited. Consequently, the actual version of the framework integrates a feature-level fusion scheme in the coordinator node.

Features used for the postures estimation with the selected data fusion technique are the following: average, energy, dynamic and static acceleration variation, kurtosys, skewness for each axis (eighteen features extracted from the wearable sensor), overall height of the monitored subject and three midpoints of 3D coordinates for points A, C and D as shown in Section 3.1.1 (ten features extracted from the ambient sensor), obtaining twenty-eight features in total.

### 3.2. Methodology for EE Quantification

**Figure 8:**
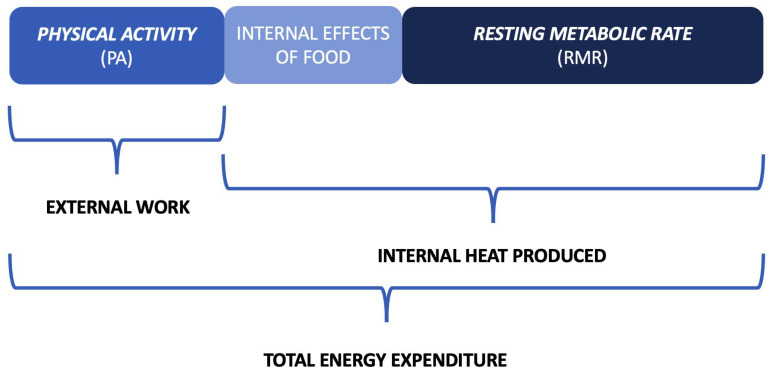
Total EE composition. It is composed of three major components: physical activity (PA), the internal effects of food and Resting Metabolic Rate (RMR).

EE can be subdivided into Resting Metabolic Rate (RMR), thermic effects of food and PA. A graphic representation of EE composition is shown in Figure 8. RMR is the quantity of energy needed to maintain body temperature, repair internal organs, support cardiac function, maintain ionic gradients across cells and support respiration. This constitutes approximately two-thirds of total EE. The second largest component of EE is required for physical work. The EE required to move the body is related directly to body weight, to the distance that weight is moved and to the state of physical fitness. Generally, EE quantification is the most reliable quantity for PA estimation. EE is normally estimated in a unit called Metabolic Equivalent of Task (MET) [45], which represents the energy (1 Kcal) or volume of oxygen (3.5 mL O_2_) consumed by a person at rest per kilogram of body weight per minute. This estimation varies for each person [46]. Consequently, one MET (the energy equivalent expended by an individual while seated at rest) and EE can be defined by the following equations:

(1) $$\begin{array}{ccc} 1\;\mathrm{MET} & = & \frac{1\;\mathrm{Kcal}}{\mathrm{kg}*h}=\frac{3.5\;\mathrm{mL}\;O_{2}}{\mathrm{kg}*\min} \end{array}$$

(2) $$\begin{array}{ccc} \mathrm{EE} & = & \frac{3.5*\mathrm{MET}*\mathrm{weight}}{200} \end{array}$$

EE unit of measurement is kilocalories burned × minute.

It is important to note that there are different approaches in the literature for quantifying MET values with respect to the specific PA performed. Generally, they are experimentally and statistically derived from a sample of persons as indicative averages, since the level of intensity could deviate from the representative experimental conditions used for the calculation of the standard MET values [47]. Table 1 reports MET values associated with postures and walking activity that were used in the present work to quantify EE, inspired by the lookup table for the equivalent MET of a series of activities reported in Table 1.

If, for example, we wanted to calculate the EE of a subject with a weight of 75 kg who walks for 18 min at a speed of 2.0 km/h (with a MET equals to 2.6 as reported in Table 1), using the above formulas, we obtain the following value:

(3) $$\mathrm{EE}\;(\mathrm{Kcals})=\frac{3.5*2.6*75}{200}*18=61.42$$

**Figure 9:**
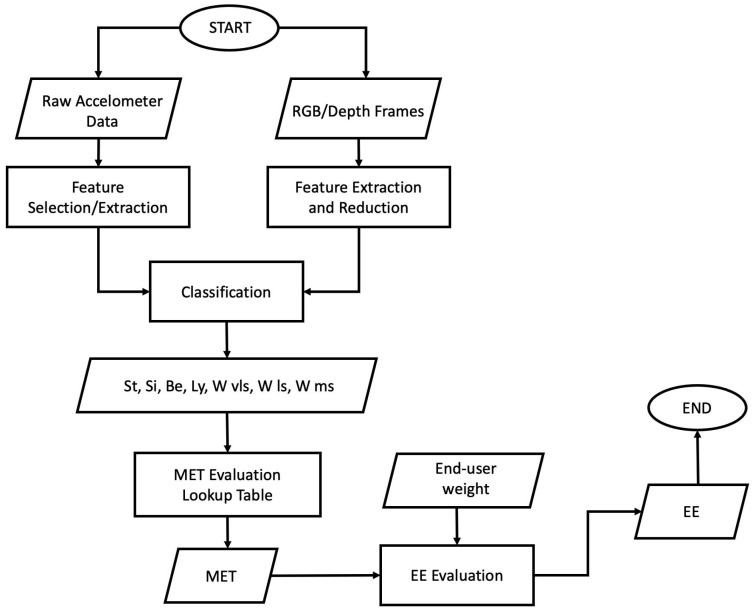
Flowchart clarifying the implemented platform’s operation. The information processed by the sensory nodes integrated in the platform classifies, after the data fusion step, four different postures and walking activities at three different speeds. Using lookup tables for MET and weight of the end-user, it is possible to quantify EE.

The flowchart and pseudocode for the designed and implemented algorithmic pipeline are shown in Figure 9 and in Algorithm 1, respectively.
**Algorithm 1** Pseudocode of the Implemented Pipeline for EE Quantification**Input wearable sensor**: $X_{t},Y_{t},Z_{t}$ (raw accelerometer data at time t)
**Input ambient sensor**: RGB/DEPTH image at time t
**Input end-user weight**: *w*
**Output**: EE.
  1: **procedure**
WearableSensor
  2:    **while** i< window_calibration_lenght **do**
  3:        $X_{cal},Y_{cal},Z_{cal}\leftarrow$calibration($X_{i},Y_{i},Z_{i}$)
  4:    **end while**
  5:    **for each** sliding window of lenght *h*
**do**
  6:        **for**
*p*
=1…h **do**
  7:           $X_{p},Y_{p},Z_{p}\leftarrow$preprocessing($X_{cal},Y_{cal},Z_{cal}$)
  8:           featureSelection/Extraction($X_{p},Y_{p},Z_{p}$)
  9:        **end for**
10:    **end for**
11:    **return** wearable features
12: **end procedure**
13: Postures ← classification(wearable features)
14: **procedure**
AmbientSensor
15:    **for each** RGB/DEPTH image at time t **do**
16:        RGB${}_{p}$/DEPTH${}_{p}\leftarrow$preprocessing(RGB${}_{t}$/DEPTH${}_{t}$)
17:        featureExtraction/Reduction(RGB${}_{p}$/DEPTH${}_{p}$)
18:    **end for**
19:    **return** ambient features
20: **end procedure**
21: Postures ← classification(ambient features)
22: **procedure**
FeatureFusion
23:    total_feature_set = wearable features + ambient features
24: **end procedure**
25: Postures ← classification(total_feature_set)
26: **procedure**
EEQuantification
27:    EE =0
28:    **for**
*i*
=1…m (minutes) **do**
29:        EE (Kcals) = EE $+\frac{3.5*\mathrm{MET}({\mathrm{Postures}}_{i})*w}{200}$
30:    **end for**
31:    **return** EE
32: **end procedure**

## 4. Results

### 4.1. Participants, Experimental Setup and Protocols

The validation was conducted in the “Smart Living Technologies Laboratory” located in the Institute of Microelectronics and Microsystems (IMM) in Lecce, Italy. Due to COVID-19 restrictions, it was only possible to validate the entire platform with 11 ageing subjects, whose characteristics are shown in Table 2.

**Figure 10:**
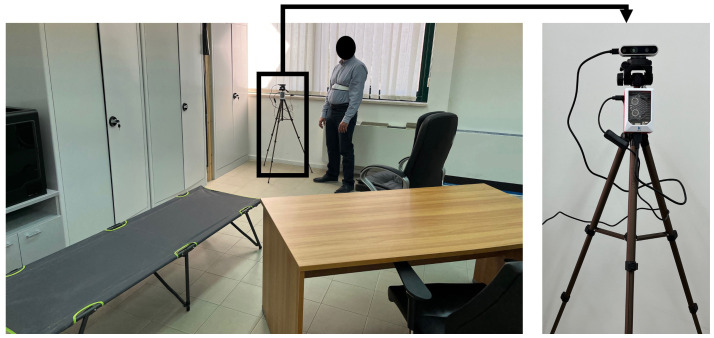
Experimental setup. The image on the left shows the laboratory area used for experimentation, the image on the right details the ambient sensory node.

The experimental design can be seen in Figure 10. A typical living environment was replicated within the laboratory, in which, there is a chair, an additional and partially visible chair positioned behind a desk, a bed and a space for walking. The performance of each detector involved in the actual version of the platform was estimated by using a common experimental setup in which the participants were asked to perform a predefined set of postures and walking activities. During such experimental sessions, data were collected simultaneously by ambient-installed camera and by the smart device worn by each participant. In order to obtain feedback on their walking velocity, each user wore a smartwatch that displays this on the screen.

To replicate as many behaviors close to reality as possible, each user performed three different data acquisition sessions, following the three protocols reported in Table 3. Sequences of static postures and walking at different speeds were varied in the three protocols so as to evaluate the classification performance of each individual sensory node, even in situations that would impair the accuracy of classification. This was carried out in order to evaluate the advantages of the integrated sensor solution.

### 4.2. Classification Performance

For each detector node, the classification performances were evaluated using accuracy and Cohen’s kappa as metrics.

In particular, accuracy is the ratio between all correctly classified samples and all samples, and is defined by the following expression:

(4) $$\begin{array}{ccc} \mathrm{Acc} & = & \frac{\mathrm{TP}+\mathrm{TN}}{\mathrm{TP}+\mathrm{TN}+\mathrm{FP}+\mathrm{FN}} \end{array}$$

where TP (True Positive) is an outcome where the model correctly predicts the positive class. Similarly, TN (True Negative) is an outcome where the model correctly predicts the negative class. FP (False Positive) is an outcome where the model incorrectly predicts the positive class. FN (False Negative) is an outcome where the model incorrectly predicts the negative class.

Since our study consider a multiclass classification problem, only the accuracy does not provide a complete overview of the classifiers’ performance. So, as shown in literature [48], Cohen’s kappa is another important performance indicator. Specifically, Cohen’s kappa is used to measure the agreement between the instance’s true label and the one predicted by the selected classifier. It is defined as:

(5) $$k=\frac{p_{o}-p_{e}}{1-p_{e}}$$

where $p_{o}$ represents the observed label and $p_{e}$ is the expected label. Cohen’s kappa always assumes values between 0 and 1. In Table 4, the correspondence between Cohen’s kappa and agreement is reported.

To reduce classification bias, a 10-cross-validation [49] was applied perturbing the training set of the classifier to randomize the original data set. Therefore, the classifier was trained for each fold using 80% of data, whereas 10% was used for validation and, at last, 10% for testing. The procedure was repeated 10 times training the classifier with a different training, validation and testing with a separated test set. It is important to highlight that the same samples do not appear in the training, validation and test sets at the same time.

Table 5 shows the performance of the two sensor nodes in accordance with the three previously described protocols. Reported values were obtained by calculating the average of the metrics considered on all users involved in the experiment. The results show that, with both sensors, an average accuracy of over 93% and a perfect agreement for kappa were achieved. A more detailed analysis demonstrates that the wearable sensor performs best in protocol 1, which contained fewer time intervals, with postures such as sitting or standing that are more difficult to distinguish with the accelerometer data. In contrast, the ambient sensor obtained the best performance in protocol 3, with fewer walking activities than in the other two protocols, because it was more difficult to estimate the different walking speeds from the images.

**Figure 11:**
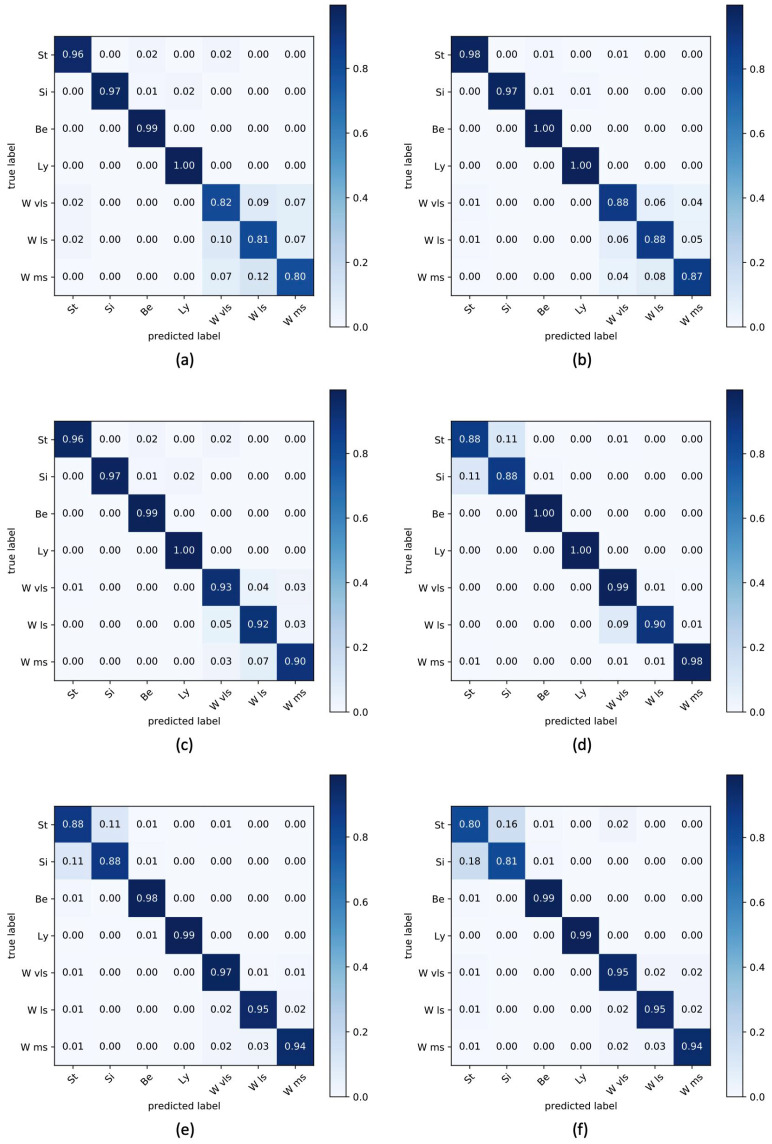
Confusion matrices for seven classes of posture and walking activities for ambient (**a**–**c**) and wearable (**d**–**f**) sensors.

In a multi-class problem, such as this study, the only metrics presented in Table 5 could not be exhaustive due to the impossibility of inspecting the separation level in terms of correct classifications among classes. To overcome this limitation, in Figure 11, the confusion matrices of the average accuracies obtaining varying sensory nodes and protocols are reported.

Since an objective of the present work is to quantify EE using information extracted from both sensory nodes simultaneously, in Table 6, the average accuracy and kappa for each considered experimental protocol are shown, considering three different ML classifiers (SVM, KNN, RF) widely used in the literature. The optimal selected parameters for each classifier were obtained through a grid search technique [50]. In particular, in SVM, we set decision_function_shape = ovo, max_iter = 50, kernel = polynomial; in RF max_depth = 30 and n_estimators = 25 were fixed; lastly, in KNN, n_neighbors = 13, metric = minkowski were considered.

The performance obtained in numerical terms proved that the accuracy of the integrated platform is always above 96%, with an increase of approximately 3% compared to the single use of the sensor nodes, confirming the goodness of the made choice.

**Figure 12:**
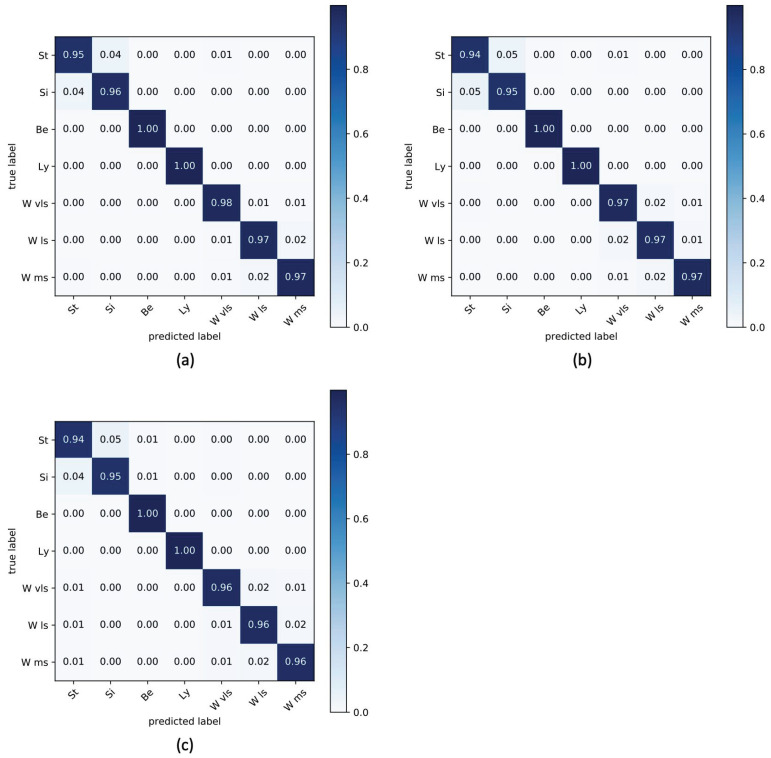
Confusion matrices for seven classes of posture and walking activities for integrated platform using RF (**a**), SVM (**b**), and KNN (**c**).

The considered classifiers were found to be equivalent with regard to their performance, with a minimum improvement obtained with RF. Confusion matrices were also considered for integrated versions of the platform. For the sake of brevity, the matrices containing average accuracies for each classifier are shown in Figure 12.

Since the goal of the present work is to automatically quantify the EE of an end-user from the sequences of postures and/or walking activities classified by the sensor nodes, it is appropriate to report the differences between an EE measurement used as ground truth and the EE quantifications obtained considering the single-sensor platform configuration and the integrated version (using the features obtained from both sensor nodes after the fusion process). The differences (reported in Table 7, Table 8 and Table 9) were estimated in terms of the relative error defined by:

(6) $$\mathrm{RE}\left(i\right)\left(\%\right)=\frac{\left|{\mathrm{EE}}_{gt}\left(i\right)-{\mathrm{EE}}_{s}\left(i\right)\right|}{{\mathrm{EE}}_{gt}\left(i\right)}*100$$

where EE${}_{gt}\left(i\right)$ is the EE used as the ground truth of the *i*-th end-user involved in the experimentation stage, whereas EE${}_{s}\left(i\right)$ is the estimated EE using each sensing technology in both single and combined modes.

For the ground truth, EE was quantified analytically from the protocols detailed in Table 3 and the respective MET reported for each posture and different walking speed in Table 1.

From the results obtained, it is evident that the EE quantification estimated by the ambient sensor node appears to be less reliable when the level of physical activity increases (more frequent walking activities), and this is due to the classification performance obtained by this detector node and reported in the previous confusion matrices. In the same way, the EE quantification estimated by the wearable sensor node has a higher relative error at the protocol execution where standing and sitting postures are most frequently present, but this result was expected given the greater difficulty in distinguishing these postures with the aforementioned sensor device.

A further conclusion from analysing the data in the three previous tables above is that, thanks to the fusion of the features for classifying postures and walking activity, the estimated EE quantification always has a relative error of less than 3.2% for each user, confirming the correctness of the algorithmic choices and demonstrating the usefulness for measurement purposes of the entire implemented platform.

Finally, the numerical results reported in the previous three tables allow us to make specific analyses of the appropriateness of the followed protocol. In fact, for example, the relative errors reported in users 7 and 10 are indicators of improper elastic band placement (and relative orientation of the Shimmer sensor). In addition, the relative errors measured with respect to the ambient sensor show an outlier performance for user 8. This difference is probably due to the physical characteristics of the subject, which may have influenced the feature extraction procedure.

## 5. Discussion and Conclusions

In the previous sections, we have described the design and implementation of a platform that provides a novel tool for the automatic quantification of EE. The advantages of the proposed solution are to be found in the involvement of heterogenous sensor technologies able to classify the same set of postures and the walking activity. The distributed architecture follows the Ambient Intelligent (AI) paradigm pursuing a series of objectives, such as assisting ageing adults in their daily life activities, detecting abnormal patterns or abnormal behavior, providing help in risky situations and monitoring specific quantities (in our case, EE) to enable ageing adults to live independently. From the usability point of view, the platform is consistent with the independent living context since it allows us to obtain an objective measure that can be analyzed offline by a doctor for subsequent clinical evaluations. A very important feature of the analyzed platform is its versatility, which stems from the consideration that it can potentially operate with any sensor/detector that is able to classify the four postures and the activity of walking. The actual version of the platform has been validated by two detectors based on sensing principles that are all compatible with the AAL scenario.

The use of the commercial devices used in this paper and a processing unit such as Raspberry makes the entire solution affordable from a cost perspective, thus ensuring its wide deployment.

Looking specifically at the obtained results, there were no substantial deviations in the detected accuracies between the different sensor types. The performance, when varying the designed protocols, is in line with the specific characteristics of the sensor node type. The ambient sensor proved more accurate in classifying static postures, whereas the wearable sensor distinguished the walking activity at different speeds better. It is worth noting that the feature set considered in the algorithmic pipelines was focused on the output posture and activity set of the present study. From the EE quantification point of view, there is a very good approximation of the measurement by the simultaneous use of the sensors.

Regarding purely software development aspects, the algorithmic pipelines, as highlighted in the previous paragraphs, were developed in Python programming language vers. 3.7. For data acquisition from the two sensor nodes, freely available libraries were used.

For the purpose of this paper, processing times were not considered to be evaluated because the output information of each sensory node was sampled at 1 s, allowing for real-time operation as well.

To the best of the author’s knowledge, exclusively the work reported in [51] considers the use of environmental and wearable sensors for EE quantification through the fusion of extracted information from heterogeneous sensors. However, differences exist with respect to our work regarding sample demographics (we have considered only ageing subjects) and employed sensory devices.

In conclusion, it is possible to highlight the following strengths of the entire system designed and implemented: (1) the algorithmic pipeline allows for objective EE quantification with multi-sensor devices that are readily available on the market, low cost and user friendly; (2) the use of heterogeneous sensors allows is to increase the acceptability level of the whole solution, as some end-users may prefer contact monitoring over non-contact monitoring (and vice versa); (3) the use of posture trends over time for EE quantification allows for the integration, in future versions of the platform, of further sensor nodes capable of reproducing the same set of features.

However, the present study has limitations, which are listed as follows. First, the number of involved ageing subjects was not very high due to the pandemic situation. Consequently, the obtained results may not be statistically consistent. In addition, the eleven subjects involved in the trial did not have mobility disorders and so it was not possible to evaluate the algorithmic framework with this type of subject. Secondly, the methodology used as ground truth does not correspond to the gold standard, i.e., indirect calorimetry. The ground truth methodology used in this paper is valid in settings where ideal conditions for EE quantification can be reproduced. Such conditions may not be present in a typical AAL environment. Thirdly, the operating ranges of the two sensor devices limit the monitoring area due to the specifications of the Bluetooth protocol integrated in the wearable sensor and the field of view and resolution of the ambient sensor. Finally, an important limitation is related to the small number of activities recognized by the sensory nodes since, within a living environment, they vary significantly, and activities that are different to walking are common.

Future work will consider the evaluation of additional commercial sensors, the development of appropriate pipelines for the recognition of a larger set of activities and, finally, the extraction of sensory data that would also allow for the assessment of the observed subject’s health status.

With respect to the latter consideration, for example, the measurement of vital parameters (such as heart rate or breath rate) during the performance of Activities of Daily Living (ADLs) could be evaluated in order to provide indications about the mood and/or stress level of the observed subject. Such information turns out to be of paramount importance when considering elderly subjects, as it heavily affects their lifestyle and leads to the onset of disorders or diseases.

## Figures and Tables

**Table 1:** Lookup table for EE through the MET values associated with the different postures and walking activity classified with the ambient and wearable sensor.

Posture	MET
Standing	1.2
Sitting	1.0
Bending	0.9
Lying down	1.0
**Walking**	**MET**
Very low speed (<1.5 km/h)	1.9
Low speed (1.5–3.0 km/h)	2.6
Medium speed (3.0–4.5 km/h)	3.4

**Table 2:** Participant characteristics. Gender, age, weight and body mass index are reported for each end-user.

Participant No.	Gender	Age (Year)	Weight (kg)	Body Mass Index (BMI)
1	MALE	67	81	27.06
2	FEMALE	71	55	23.19
3	FEMALE	70	59	23.04
4	MALE	70	75	27.54
5	MALE	65	66	21.30
6	FEMALE	68	61	26.40
7	FEMALE	68	52	18.42
8	MALE	69	83	31.23
9	FEMALE	65	54	20.83
10	MALE	69	78	28.65
11	MALE	73	70	23.66

**Table 3:** Used protocols. Each activity is reported with the following acronyms: St = standing; Si = sitting; Be = bending; Ly = lying down; W vls = walking very low speed; W ls = walking low speed; W ms = walking medium speed.

Protocol 1		Protocol 2		Protocol 3	
**Posture/Action**	**Dur (s)**	**Posture/Action**	**Dur (s)**	**Posture/Action**	**Dur (s)**
W vls	30	W vls	60	Si	30
St	60	Ly	30	St	30
W ls	30	St	30	W ls	30
Si	90	Be	30	Ly	60
W ls	30	W ls	30	St	30
St	30	Ly	60	Be	30
W ms	30	W ls	30	W vls	30
Be	30	St	60	Si	60
W vls	60	W ms	60	St	30
Si	30	Si	30	W vls	30
Total dur (m)	7		7		6

**Table 4:** Cohen’s kappa vs. agreement.

Cohen’s Kappa	Agreement
k<0.20	slight
0.21≤k<0.40	fair
0.41≤k<0.60	moderate
0.61≤k<0.80	good
0.81≤k≤1.00	perfect

**Table 5:** Classification performance for ambient and wearable sensors not considering data fusion and at three varying different protocols.

Sensor Node	Protocol 1		Protocol 2		Protocol 3	
	Accuracy	k	Accuracy	k	Accuracy	k
Ambient	0.905	0.890	0.941	0.931	**0.953**	0.945
Wearable	**0.957**	0.950	0.939	0.928	0.919	0.906

**Table 6:** Classification performance for ambient and wearable sensors considering data fusion and at three varying different protocols.

Classifiers	Protocol 1		Protocol 2		Protocol 3		Avg Accuracy
	Accuracy	k	Accuracy	k	Accuracy	k	
RF	0.984	0.978	0.972	0.931	0.973	0.969	0.976
SVM	0.980	0.968	0.970	0.958	0.967	0.942	0.972
KNN	0.975	0.956	0.968	0.960	0.963	0.937	0.968

**Table 7:** Evaluation of RE (expressed in %) for EE quantification using ambient sensor, wearable sensor and the integrated solution (Protocol 1).

User	EE_*gt*_ (Kcal)	RE (%)		
		Ambient	Wearable	Integrated
1	16.15	9.4	3.5	2.7
2	14.96	7.6	4.1	3.0
3	13.16	8.1	3.7	2.9
4	16.55	7.7	3.4	2.8
5	15.56	6.6	4.4	2.5
6	13.96	7.8	4.0	3.0
7	10.97	9.0	7.7	3.2
8	11.77	13.3	3.9	2.5
9	12.16	8.1	3.5	2.2
10	10.37	8.0	7.2	2.4
11	10.77	8.9	4.0	2.7

**Table 8:** Evaluation of RE (expressed in %) for EE quantification using ambient sensor, wearable sensor and the integrated solution (Protocol 2).

User	EE_*gt*_ (Kcal)	RE (%)		
		Ambient	Wearable	Integrated
1	18.78	6.8	4.5	2.5
2	17.39	6.0	4.7	2.7
3	15.30	5.3	4.2	2.8
4	19.24	6.1	4.9	3.0
5	18.08	5.8	5.4	2.6
6	16.23	6.0	5.3	3.1
7	12.75	7.2	7.9	2.9
8	13.68	11.1	4.6	2.2
9	14.14	7.9	5.0	3.0
10	12.05	6.1	9.5	2.5
11	12.52	6.2	5.3	2.6

**Table 9:** Evaluation of RE (expressed in %) for EE quantification using ambient sensor, wearable sensor and the integrated solution (Protocol 3).

User	EE_*gt*_ (Kcal)	RE (%)		
		Ambient	Wearable	Integrated
1	11.26	3.4	7.4	2.1
2	10.43	4.1	7.7	2.2
3	9.18	3.9	6.3	3.1
4	11.54	4.2	6.8	2.7
5	10.85	3.9	7.6	2.4
6	9.73	4.1	8.0	2.8
7	7.65	4.2	10.4	3.2
8	8.20	8.0	6.9	2.5
9	8.48	3.3	6.6	2.5
10	7.23	4.0	11.8	2.8
11	7.51	3.5	7.7	2.7

## Data Availability

The data presented in this study are available on request from the corresponding author. The data are not publicly available due to restrictions (they contain information that could compromise the privacy of research participants).
